# Sexuality and ageing: A mixed methods explorative study of older adult’s experiences, attitudes, and support needs

**DOI:** 10.1177/03080226231164277

**Published:** 2023-04-12

**Authors:** Amelia Portellos, Claire Lynch, Annette Joosten

**Affiliations:** School of Allied Health, Australian Catholic University, Melbourne, VIC, Australia

**Keywords:** Sexual health, intimacy, older adults, supports

## Abstract

**Introduction:**

Sexuality contributes to health and wellbeing, but it is often overlooked as an important occupation for older adults. Older adult studies focus on decreased participation and functioning in sexual acts. This study aimed to obtain perspectives and explore understandings and experiences of sexuality and of support needs, for typically ageing adults.

**Method:**

Seventy-five older adults aged 60 years and older participated in the online cross-sectional survey. Participants were primarily from Australia, the United States, and the United Kingdom. Closed questions were analyzed using descriptive and inferential statistics, and reflexive thematic analysis was used for open questions.

**Results:**

Throughout the ageing process, older adults continued to value their sexuality, expression, desire, and identity, though meanings and participation changed. Changes and challenges were overcome through openness, exploration, curiosity, valued relationships, and adaptations to ways of doing. Older adults identified being dissatisfied with current sexual health supports, resources, and services.

**Conclusion:**

Healthcare professionals need to develop intervention knowledge to address diverse needs, and better support older adults in sexuality through open discussion, addressing psychological, social, and health concerns as part of everyday practice. The development of standardized assessments and resources with consumer collaboration will ensure resources address multifaceted components of sexuality, and older adults’ needs.

## Introduction and literature review

Environmental, social, medical, and economic expansion have resulted in an increase in the ageing population over the past 40 years (UN, 2017). This has placed an onus on understanding ageing processes to inform and enhance older adults’ health outcomes (Sowa et al., 2016). One aspect of health research and policy often overlooked for typically ageing populations is sexuality. Sexuality is an ever-changing and individualized concept (Fileborn et al., 2015). Sexuality encompasses sexual activity, sexual identity, orientation, desire, companionship, intimacy, sexual attitudes, and health (Chen et al., 2017; Hyland and McGrath, 2013; WHO, 2006).

Sexuality definitions are broad and nuanced, they rely on societal and cultural norms, and the role individuals have within a relationship, or community (Fileborn et al., 2015, 2017a). Sexuality is linked to who people are (physically, psychologically, and spiritually), what people do, and how they do it, to bring importance to their lives (Lynch and Fortune, 2019; Ménard et al., 2015). In this way, sexuality fits within a biopsychosocial understanding of meaningful occupation, centralizing varied influences on sexual identities, roles, and activities (DeLamater and Koepsel, 2015; McGrath and Lynch, 2014). This multidimensional view of sexuality has emphasized the holistic formation of sexuality as an occupation (Lynch and Fortune, 2019; Sakellariou and Algado, 2006) and has highlighted its contribution to quality of life (Freak-Poli and Malta, 2020).

Previous research reported sexuality, intimacy, and relationships as integral to an individual’s health and wellbeing, with illness, injury, and environmental factors negatively affecting sexual participation (Fileborn et al., 2017c; Heywood et al., 2019; Lodge & Umberson, 2012). Reporting of the complications related to sexuality as people age has largely focused on sexual functioning concerns (Lindau et al., 2007). Suggestions from two large quantitative studies (Laumann et al., 2009; Lyons et al., 2017) and three qualitative studies explicitly highlighted the continuing importance of other aspects of sexuality for older persons (Fileborn et al., 2015; Sandberg, 2013; Stahl et al., 2019). Older adults in two qualitative studies maintained their sexuality by transparently communicating needs with partners, being empathetic, and adaptable to changing health and relationships (Lodge and Umberson, 2012; Ménard et al., 2015). Little is known about broader experiences of adapting to changes for older adults, especially unpartnered, non-heterosexual, and non-cisgendered populations.

Explicitly discussing sexuality is not new for many current older adults in the Baby-Boomer population of Western countries. Influenced by the social and political climate of the 1960s and 1970s, this population in their youth greeted and destigmatized sexuality and challenged gender roles to bring about more socially progressive views (Rahn et al., 2020). Literature suggests that as people age, community attitudes become less accommodating, and healthcare systems are less responsive to sexual needs (Fileborn et al., 2017b; Schaller et al., 2020). The consequences of this indicate that while many older adults across Europe and the United States have experienced participation barriers in sexual occupations, approximately only half reported seeking formal support (Hinchliff et al., 2019; Laumann et al., 2009; Lindau et al., 2007).

Barriers have prompted older adults to self-educate through alternative sources (Fileborn et al., 2017d; Hinchliff et al., 2019). Limitations were found in education and resources, which prioritized physical changes and dysfunction in sexual activity over multifaceted components of sexuality (Fileborn at al., 2015; Syme et al., 2019). There is very limited research on contributors to participation, satisfaction, and expressions of sexuality through ageing (Syme et al., 2019). Therefore, the extent to which holistic variances in ageing and societal changes affect one’s engagement with sexual occupations, identity, and expression are largely unknown.

Priorities of this study were to understand sexual attitudes and changes throughout the lifespan and the importance and meaning of sexuality from the perspective of typically ageing older adults. The study aimed to understand the unique experiences of sexuality for typically ageing older adults by exploring what sexuality means and how important it is. We sought to understand the changes, barriers, and enablers ageing populations experience in maintaining their sexuality, the diversity of supports they may need, and how these should be addressed in occupational therapy contexts. As occupational therapists, we were interested in how older people considered sexuality, its relationship to health and wellbeing and identity and what we could learn about better supporting sexuality. The study additionally aimed to identify if differences were experienced across demographic groups of older adults and to what level “typically ageing” adults experienced changes and barriers as compared to those who lived with physical or psychological health conditions.

## Materials and methods

### Research design

An explanatory cross-sectional mixed methods design was used to obtain descriptive data from the sample and provide a general population snapshot (Schofield and Forrester-Knauss, 2017). A population-based survey was used to collect quantitative and qualitative data (Portney and Watkins, 2014; Qualtrics, 2019) on the experiences and opinions of older adults toward sexuality, this explorative approach was deemed suitable to gain a better understanding. Given the personal nature of sexuality, an anonymous survey was decided upon to ensure participants felt comfortable discussing their unique experiences. This study was approved by a Human Research Ethics Committee (2020-202EAP).

### Participants and recruitment

Inclusion criterion was adults 60 years, and older who consented to participation in the study, this included older adults across diverse genders, age ranges, sexual orientation, relationship statuses and country of residence. This age was decided as it is commonly used to define “older age” by senior rights and government services (Health.Vic., 2020). Limited exclusion criteria were implemented to increase participant uptake. Individuals with disabilities and diversity factors were not excluded; however, these influences were considered in the demographic sections of the survey, and their impact was to be considered in analysis. Research participants were recruited using a multitude of methods, through advertising the survey on the researchers’ professional networks and through consultation with consumer specific agencies (The Spicy Boudoir and the Australian Association of Gerontology). Participants from Australia, the United Kingdom, Ireland, and the United States completed the survey. To increase the sample size and facilitate snowball sampling, advertisements with URL and QR links were placed on social media, in community centers and retirement villages in the researcher’s localities. No direct contact was made between participants and researchers to ensure confidentiality and limit risk of bias. Participants were advised at the start of the participant information letter that submission of the survey assumed informed consent. Paper-based surveys were available and distributed in community centers as a choice for older adults.

### Measures

Data were collected using an online survey developed originally by the research team. Existing sexuality in ageing measures like the Ageing Sexual Knowledge and Attitudes Scale were reviewed during development (White, 1982). Existing tools focused primarily on changes to sexual activity, not holistic understandings of sexuality as an occupation; therefore, the survey was developed to capture broader understandings and experiences of sexuality including identity, expression, and dynamics in healthcare.

The survey comprised four sections: (a) demographic information; (b) meanings, attitudes, and experiences of changing sexuality with age; (c) perceptions about enablers and barriers; and (d) supports required to maintain sexuality. The survey was piloted with four older adults in February 2021, and amendments were made to various closed questions allowing a “prefer not to answer” option, to reduce participant distress and non-response bias. Additional amendments ensured language used in the survey captured experiences outside of heteronormative constructs (e.g. “sexual activity” replaced “intercourse”). A forced answer feature for closed questions was utilized to ensure there were no missing data.

**Figure 1.**
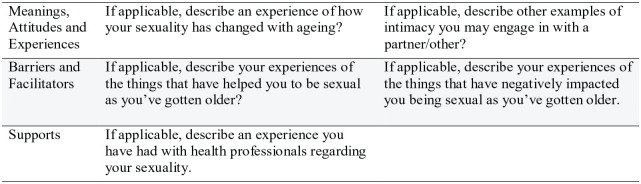
Qualitative questions examples.

Closed and Likert-type style questions were included in the survey to measure demographic information, attitudes, frequencies, and extent of agreement (Portney and Watkins, 2014). As closed questions limit nuanced responses and sexuality is inherently encapsulated within an individual’s identity, several open questions were utilized to gain in-depth responses pertaining to meaning and experiences (Portney and Watkins, 2014). As an explanatory mixed-method study, the open questions were designed to provide more in-depth explanation about the closed question responses about the individual and complex nature of sexuality for the participants, who were asked to describe examples of intimate occupations, their experiences of sexuality changing, influences on their sexuality, and their involvements with health professionals (Figure 1).

### Quantitative analysis

Participants’ data were downloaded and exported into SPSS software (version 27; IBM, 2020). Survey data were analyzed, producing descriptive statistics to outline the sample demographic details and survey responses (Portney and Watkins, 2014). Inferential statistics were used to identify differences in attitudes or expression, as concurrent with variables such as age, gender, and sexual orientation. Correlations measured the strength of relationships between survey responses and demographic variables.

The Mann–Whitney *U*-tests were conducted to explore if complications affecting sexuality were experienced comparatively for “typically ageing” individuals and those with disabilities, physical and mental health conditions. This was measured to understand the extent to which ageing itself indicated changes to sexuality. The Kruskal–Wallis tests were used to identify differences in responses when there were three or more groups in the variable (e.g. sexual orientation). Based on the literature, demographic variables were expected to show differences in frequency of sexual activity, sexuality meaning, and barriers to accessing supports. A Pearson Correlation Coefficient measured the strength of the relationship between sexual orientation and therapeutic relationship experiences.

### Qualitative analysis

Qualitative results were analyzed to gain richer insights into the lived experience and meaning of sexuality through ageing. The process of reflexive thematic analysis was used to analyze open-ended questions (Braun and Clarke, 2019). Steps including familiarization and coding of the responses, generating initial themes, and reviewing and defining the themes were implemented (Braun and Clarke, 2019). The responses to open-ended questions were read and coded by each of the three research team members, this was followed by collaborative discussion, and reflection among the team. The incorporation of the researchers’ theoretical assumptions about sexuality as an occupation linked to health, wellbeing and identity were identified, and biases were transparently discussed, with the researchers coming from three generations, two female and a nonbinary researcher. Following this, developing themes were considered and reviewed before overarching central organizing themes were developed based on the researchers shared patterns of meaning (Braun and Clarke, 2019). This iterative and reflexive process ensured triangulation among the research team and with the closed question responses, ensuring the developed themes were credible based on the data collected and trustworthiness was further ensured using audit trails to enhance dependability of the data (Nowell et al., 2017).

## Results

### Participants

A total of *n* = 127 participants completed the online survey. On examination of the responses, and in consultation with Qualtrics administration, 52 surveys were deemed duplicates or fraudulent attempts based on Qualtrics monitoring settings; therefore, 75 responses were included in the analysis. Of the participants, 56% were female, 41% were male, and 2.7% were nonbinary. The majority participants were between 60 and 70 years old; however, over one-third of participants were over 71 years old. Heterosexuality accounted for 75% of participants’ sexual orientations, other common orientations included bisexual (12%), asexual (4%), gay, lesbian, or queer (3% respectively). Over half of the respondents were married or partnered (57%) or in open relationships (7%). Single participants accounted for 15%, and a further 13% reported they were separated, divorced, or widowed. Participants largely resided in their own homes or lived with family. Of the 75 respondents, 15 individuals reported living with a disability (20%).

## Quantitative results

### Sexuality meaning and frequency

Participants were asked to define what “sexuality” meant for them, the most common responses were pleasure (physical: 69% and mutual: 68%), relationships (63%), and sex (63%). Frequency of sexual activity was reported as occurring at least weekly for 48% of participants, whereas 27% of participants rarely or never engaged in sexual activity (Mdn = 3, interquartile range (IQR) = 4). A significant difference (*p* = 0.003) was found using a Kruskal–Wallis test to identify differences between activity frequency and varied relationship types. Participants in married, open, or partnered relationships indicated higher sexual frequency (once a week) than those who identified as single, widowed, or separated (once a month to never). Desired sexual frequency was more than actual sexual activity in all relationship types (Mdn = 2, IQR = 2).

### Sexuality changes, satisfaction, and importance

Respondents were asked if the meaning of sexuality had changed through ageing, with 67% agreement. A Mann–Whitney *U*-test analysis was used to investigate whether physical or mental health conditions affected one’s sexual expression as they aged. This showed a significant difference between mental health experiences and difficulties in expression over time (*p* *=* 0.020).

Participants reported varied satisfaction with their overall sex lives (see Table 1). The importance of sexuality was measured by addressing common components of sexuality: sexual activity and “being a sexual being.” Thirty-five participants stated maintaining sexual activity was very/extremely important (47%), and *n* = 37 (49%) participants stated slightly/moderately important. Being a sexual being was rated very/extremely important by 45% and slightly/moderately by 51% of participants. Important components of sexuality were additionally measured (response rates are presented alongside experienced changes in Table 1). Differences were measured between gender, and important aspects of sexuality, showing a statistically significant difference (*p* *=* 0.045) for the “feeling desired and sexual” component. Post hoc analysis showed differences were found for nonbinary participants who rated this of higher importance (*p* ⩽ 0.001).

**Table 1.** Likert-type responses for changes, satisfaction, and importance.

	1	2	3	4	5	Median	IQR (Q_3_–Q_1_)	*n*
	Strongly disagree *n* (%)	Disagree *n* (%)	Neutral/unsure *n* (%)	Agree *n* (%)	Strongly agree *n* (%)			
Sexuality has changed its meaning as I have aged.	2 (2.7)	11 (14.7)	12 (16.0)	38 (50.7)	12 (16.0)	4	1	75
Expressing myself sexually has changed as I have aged.	2 (2.7)	9 (12.0)	16 (21.3)	36 (48.0)	12 (16.0)	4	1	75
It is harder for me to feel comfortable expressing my sexuality now that I am older.	17 (22.7)	20 (26.7)	20 (26.7)	18 (24.0)	0 (0.0)	3	1	75
Overall, I feel satisfied with my sexuality and sex life.	4 (5.3)	19 (25.3)	20 (26.7)	22 (29.3)	10 (13.3)	3	2	75
Having “sex” is the most important part of sexuality for me.	5 (6.7)	24 (32.0)	31 (41.3)	14 (18.7)	1 (1.3)	3	1	75
*Feeling desired and sexual is the most important part of sexuality for me.	1 (1.3)	9 (12.0)	16 (21.3)	42 (56.0)	7 (9.3)	4	1	75
Feeling loved and having roles in my relationship/s is the most important part of sexuality for me.	1 (1.3)	3 (4.0)	11 (14.7)	46 (61.3)	14 (18.7)	4	0	75
I want to continue being a sexual person for as long as I live.	2 (2.7)	3 (4.0)	13 (17.3)	26 (34.7)	31 (41.3)	4	1	75

IQR: interquartile range.

* The Kruskal–Wallis analysis.

### Barriers and facilitators to maintaining sexuality

When asked if respondents had experienced ageism, which had affected their independent choices and sexuality, 41% of them were neutral, and 37% of them disagreed. Participants similarly disagreed across all questions in the domain, which assessed older adults’ experiences of and reactions to societal attitudes (see Table 2). There was mixed opinion about barriers to discussing and accessing support for sexual health concerns. Analysis found a significant difference in barriers was experienced by those renting compared to those who owned their home (*p* = 0.014).

**Table 2.** Likert-type responses for barriers and facilitators toward sexual expression.

	1	2	3	4	5	Median	IQR (Q_3_–Q_1_)	*n*
	Strongly disagree *n* (%)	Disagree n (%)	Neutral/unsure *n* (%)	Agree n (%)	Strongly agree *n* (%)			
I have experienced ageism relating to my independent choices and sexuality	9 (12.0)	19 (25.3)	31 (41.3)	16 (21.3)	0 (0.0)	3	1	75
Social attitudes or judgment concerning older people not engaging in sex impacts how I feel about my sexuality.	10 (13.3)	39 (52.0)	16 (21.3)	10 (13.3)	0 (0.0)	2	1	75
Sometimes I feel like the media/society makes me feel like I should not be having sex.	11 (14.7)	31 (41.3)	17 (22.7)	14 (18.7)	2 (2.7)	2	1	75
I feel like I should not be interested in or having sex now that I am getting older.	31 (41.3)	28 (37.3)	6 (8.0)	10 (13.3)	0 (0.0)	2	1	75
I feel embarrassed talking to a health professional about sex/sexuality.	14 (18.7)	27 (36.0)	16 (21.3)	15 (20.0)	3 (4.0)	2	1	75
*I feel like there are a lot of barriers to me discussing and accessing support for my sexual health concerns.	2 (2.7)	25 (33.3)	27 (36.0)	18 (24.0)	3 (4.0)	3	2	75
I feel like attitudes toward sexuality for older people have changed in the past 20 years.	2 (2.7)	3 (4.0)	18 (24.0)	47 (62.7)	5 (6.7)	4	1	75
I feel like people want to listen to me when I talk about my sexual/health concerns	2 (2.7)	17 (22.7)	40 (53.3)	13 (17.3)	3 (4.0)	3	1	75
I would feel comfortable talking to friends and family about physical sexual health concerns (e.g. erectile dysfunction, vaginal dryness)	9 (12.0)	21 (28.0)	23 (30.7)	17 (22.7)	5 (6.7)	3	2	75
I would feel comfortable talking to friends and family about emotional sexual health concerns (e.g. desire changes and relationship issues)	9 (12.0)	19 (25.3)	20 (26.7)	23 (30.7)	4 (5.3)	3	2	75
I feel confident that I can talk to my current sexual partner/s about sexual health and STIs.	5 (6.7)	10 (13.3)	15 (20.0)	26 (34.7)	19 (25.3)	4	2	75

IQR: interquartile range.

* The Kruskal–Wallis analysis.

Participants generally perceived attitudes toward sexuality for older people had become more accepting over the past 20 years. Other facilitators were measured by exploring how and where participants sought support (family, friends, and partners; Table 2).

### Formal supports and resources

In the 2 years prior to the survey, 57% of respondents stated they had never visited a healthcare professional (HCP) in relation to a sexual problem, similarly 55% stated HCPs had never discussed sexuality, but 43% of participants described experiencing a sexual health concern over the past 2 years. Participants’ likelihood of seeking formal support by discussing concerns with professionals found, 49% of them were likely, 29% of them felt unsure, and 21% of them were unlikely (Mdn = 3, IQR *=* 1).

Forty participants (53%) reported good therapeutic relationships with HCPs which increased comfort in addressing sexual health concerns. Differences were analyzed between therapeutic relationships and gender, age, or sexual orientation. Sexual orientation showed the only significant difference (*p* = 0.035), detailing that asexual participants experienced fewer positive relationships compared to other participants. A Pearson’s correlation coefficient and two-tailed significance test revealed a weak positive correlation and high statistical difference (*r* = 0.31, *p* *=* 0.007), indicating that asexual older adults were likely to have less encouraging therapeutic relationships. Overall, participants reported uncertainty or dissatisfaction with current supports and resources. However, 49% of participants reported confidence in their current knowledge of sexuality and competence when asked to theorize accessing resources (56%; Table 3).

**Table 3.** Likert-type responses for supports.

	1	2	3	4	5	Median	IQR (Q_3_–Q_1_)	*n*
	Strongly disagree *n* (%)	Disagree *n* (%)	Neutral/unsure *n* (%)	Agree *n* (%)	Strongly agree *n* (%)			
*^I have a good relationship with my regular health professional and would feel comfortable talking about sexual health concerns.	3 (4.0)	8 (10.7)	24 (32.0)	25 (33.3)	15 (20.0)	4	1	75
If I was experiencing a physical health concern impacting my sexuality, I would seek medical/healthcare assistance.	3 (4.0)	12 (16.0)	13 (17.3)	32 (42.7)	15 (20.0)	4	1	75
If I was experiencing a mental health concern impacting my sexuality, I would seek medical/healthcare assistance.	2 (2.7)	7 (9.3)	17 (22.7)	34 (45.3)	15 (20.0)	4	1	75
I feel satisfied with the amount of education/support I have seen or accessed regarding sexuality for older people.	3 (4.0)	19 (25.3)	32 (42.7)	19 (25.3)	2 (2.7)	3	2	75
I feel like there are lots of supports out there for older people wanting to learn and understand their changing sexuality.	9 (12.0)	24 (32.0)	29 (38.7)	12 (16.0)	1 (1.3)	3	1	75
I feel like I have lots of knowledge and understanding about changes to my sexuality.	3 (4.0)	11 (14.7)	24 (32.0)	27 (36.0)	10 (13.3)	3	1	75
If I had a sexual health problem I would know where to access resources to support me.	4 (5.3)	6 (8.0)	23 (30.7)	23 (30.7)	19 (25.3)	4	2	75

IQR: interquartile range.

* The Kruskal–Wallis analysis.

^ The Pearson correlation coefficient.

When participants were asked to choose supports that would enable their sexuality, the most common responses included “online resources” (64%), “healthcare professionals opening up a dialog” (51%), and “age-specific sexual health service centers” (40%).

## Qualitative results

**Figure 2.**
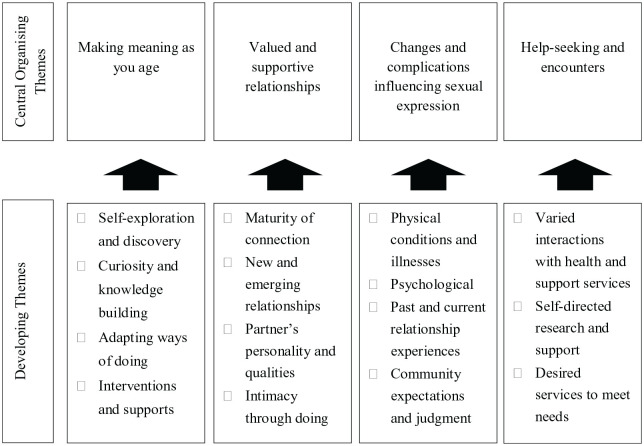
Refinement of qualitative themes.

Four central organizing themes were developed and provided richer understanding of the older adults’ quantitative responses: (a) making meaning as you age, (b) valued and supportive relationships, (c) changes and complications influencing sexual expression, and (d) help-seeking and encounters (Figure 2).

### Theme 1: Making meaning as you age

Participants detailed their experiences understanding, accepting, and adapting attitudes and practices to make meaning of their sexuality and identity. They explained participating individually and, in their relationships, with support from informal, and formal sources to overcome various changes and challenges. For some, although difficulties had changed outward expressions, their sense of being sexual remained “. . . although I had no thoughts at all about having a new partner or having sex, I never felt that I had lost my femininity as I aged” (female, heterosexual, and partnered, 60–70 years old).

Several participants explained an increase in “knowing” and “understanding” themselves, and their needs as they aged, which in turn made them more confident. “I am less shame-bound, more confident, more participatory, and more present than I used to be” (male, heterosexual, and polyamorous partnered, 60–70 years old). Exploration of self, saw some older adults’ sexual identities evolve and reject stereotyped sexual and relationship roles, which they would previously adhered to:

I came out as lesbian at 40 after being only with men. I then identified as queer after realizing an attraction to trans-men and non-binary people (AFAB), I studied sexology and learnt more, I came out as poly at 46 and non-binary at 57. (Nonbinary, queer, and partnered, 60–70 years old)

Self-exploration and openness were seen as facilitators to sexual ageing. “I’m a lot more free now to entertain my masculine side. For instance, during sex, I pretend to have a penis and penetrate my partner” (female, heterosexual, and single, 71–80 years old).

Many recognized that as they aged, they became more curious about their sexuality. Single or repartnered participants described curiosity in connections with new people, and experiences. Long-term partnered adults considered curiosity as deepening intimacy and finding activities to prolong positive sexual experiences. Both groups developed sexuality and ageing meanings through informal discussions with peers, self-determined knowledge building, and self-exploration. Naming authors including Joan Price, Esther Perel, and Emily Nagoski, some participants stated how progressive societal views and resources had contributed to maintaining open-minded attitudes toward their sexuality and identity as they aged.

Less emphasis on penetrative sex and a focus on other practices were described by many. These were not overtly recognized as “sexuality,” rather they were categorized as “intimacy.” Communication was seen as a key component to maintaining intimacy. Participants described “dinner dates, films, concerts, holding hands, dancing, cuddles, complimenting” and other mundane experiences of life as having prolonged their sexuality, “. . . sleeping naked together, cooking and eating meals, sharing the same interests, taking journeys in the car . . . watching a movie, looking after the grandkids all these activities have an intimacy to them if you so desire” (female, heterosexual, and partnered, 60–70 years old).

Sexual practices were maintained through proactivity to trial interventions to overcome changes and barriers, adaptations to acts of doing, and communication in partnerships. Participants described medical interventions as assisting in penetrative sex, but that they reduced spontaneity, and negative side effects that led some to try alternative medicines, and practices. “I’m now taking a plant estrogen; it has heightened my sexuality in many ways. It’s Kacip Fatimah. Whoa, this stuff is great that combined with pelvic PT is bringing back my youthful vagina, sensation, and sex drive” (female, heterosexual, and single, 71–80 years old). “The occasional use of MDMA heightens the experience of uninhibited physical and enthusiastic intimacy” (female, heterosexual, and partnered, 71–80 years old).

### Theme 2: Valued and supportive relationships

Older adults described that valued and supportive relationships developed as relationships matured leading to increased understanding, comfort, and support. “My partner and I have been together for 48 years. After difficult early years when we hardly knew each other, we came through and grew together. It gets really good after about 30 years!” (male, heterosexual, and married, 71–80 years old).

Many who had experienced negative past relationships, and lack of alignment of values with previous partners sought openness to new experiences and developed assurance about their wants in a relationship. Positive new relationships were influenced by a commonality of past experiences, value alignment with new partners, and a shared sense of discovery:

Now that I do have a partner who values me . . . and tells me regularly that he loves the way I look, dress, act, and he’s proud to be with me. It’s a lovely and positive change at this end of life to feel and act as I/we did when I was in my 20’s. (Female, heterosexual, and partnered, 60–70 years old)

Valued relationships were shaped by partners who were encouraging, curious, and understanding. Connectedness was experienced when older adults deliberately made time for each other, openly communicated, and relished intimate moments. Participants described partnered acts that emphasized intimacy, sustained their ageing sexuality included sleeping naked together, using vibrators, dildos, mutual masturbation, cuddling, kissing, oral sex, and manual sex. Some participants described alternative sexual exploration, non-monogamous and group sexual acts as facilitating their sexuality.

### Theme 3: Changes and complication influencing sexual expression

Participants reported that age-related difficulties including hormonal changes, erectile difficulties, and increasing frailty were common complications, but past relationships and experiences, generalized physical, and mental health conditions also influenced changes to sexuality.

The impact of physical and psychological changes varied for participants, with some detailing a cessation of sexual activity and desire, others specified “a slowing down” process, and some discussed a consequential loss of sexual self-expression and confidence. “Not being as confident of my body and feeling older makes me more hesitant to express my sexuality” (female, heterosexual, and single, 60–70 years old). Many highlighted the interrelated nature of physical, psychological, and sexual health and wellbeing. “(An) insertion of miniarc sling to prevent bladder leakage led to persistent pain in intercourse; any desire for sex has disappeared; I feel asexual” (female, heterosexual, and married, 60–70 years old).

Previously partnered participants described how relationship experiences and grief had affected their sexuality, “I’ve been celibate for so long and haven’t been with anyone for 20 years. I feel embarrassed at the thought of being sexual with someone. I feel unattractive” (female, heterosexual, and widowed, 71–80 years old). Even those who reported feeling positive about their current sexuality, detailed how negative past relationships had impacted desire. “My long-term partner was aggressively verbally abusive so sexual relations with him ceased around 2002. He died in 2008 and I had no desire for a sexual relationship until I met my current partner” (female, heterosexual, and partnered, 60–70 years old).

Some participants described how different expectations and needs in their relationships had created challenges to sexuality “I’ve dated a couple of guys in their ‘60’s and they want companionship more than sex. They want wifey to look after them (yawn)” (female, heterosexual, and single, 60–70 years old). Family and HCPs were often considered judgmental toward participants’ sexuality, older adults described feeling unsupported and not understood by those around them. “Family are not interested in hearing about me wanting to be sexual. My son is estranged from me because of me dating after my marriage with his dad broke down” (female, heterosexual, and single, 61–70 years old).

### Theme 4: Help-seeking and encounters

Most participants reported unsatisfactory service from General Practitioner (GP) and HCPs regarding their sexual health. Typically, they felt professionals lacked openness, were not understanding or empathetic, rarely initiated sexual conversations, and were limited in their knowledge of ageing sexuality. Those who saw specialized services such as gynecologists and Sexually Transmitted Infection (STI) services felt more supported in their interactions and reported positive sexual health but described difficulty in finding specialist services. Predominantly many still felt unsupported. “Personally, I advocate for myself with health professionals, and they accept this. They never bring it up if I don’t though” (female, heterosexual, and partnered, 71–80 years old).

Many reported that knowing what to expect from GPs influenced whether they sought support from generalized services or self-researched instead. One person highlighted what had been lacking and what desired supports needed to look like:

. . . my GP isn’t about to ask me how I’m doing sexually . . . They have never, ever asked! When I went to a gyn recently he gave me estrogen cream for my late-in-life wish to return to having sex and NEVER mentioned pelvic PT . . . Those docs are still in the dark ages!!! (Female, heterosexual, and single, 71–80 years old).

Older adults want health services and professionals to have more openness in discussing sexuality, greater understandings of the holistic contributors to ageing sexual health, knowledge of alternative medicines and therapies, psychosocial supports, and more standardized approaches to addressing sexuality in practice. “I had to bring up the subject of erectile dysfunction. It would have helped a lot if it was just a standard question like blood pressure is” (male, heterosexual, and married, 71–80 years old).

## Discussion and implications

This study explored sexuality, practices, and attitudes of older adults as they aged to understand their experiences and identify supports for addressing sexuality in healthcare. Findings illustrate that many older people were still frequently engaged in sexual activity, and almost all emphasized wanting to be a “sexual being” for the remainder of their lives. This was consistent with existing literature detailing the importance of sexuality throughout the ageing process (Fileborn et al., 2015; Lyons et al., 2017). The findings, particularly the qualitative responses richly reported older adult’s diverse sexual identities, desires, and intimate practices, which extended on previous literature. Life-long experiences changed, sometimes enhanced and other times challenged sexuality. Age alone was not the only contributor to sexual identity, attitudes, meaning, participation, and importance shared in this study, instead varied lived experiences relating to past and current relationships, experiences of illness, openness to new learning, and other individualized components also influenced the way participants engaged with their sexuality.

An important contribution of this study was the representation of non-cisgendered and non-heterosexual older adults (16% of participants), as their experiences and insights were often underrepresented in research on older adults’ sexuality. Many of them reported that they discovered or started to live their identities in their mid-to-later life, overcoming societal attitudes and stigma. Interestingly, there was a common experience among bisexual, queer, and pansexual participants, that exploration and coming into oneself had led to more confidence, freedom, greater interest, desire, and knowledge in their sexuality, which were seen as positives to ageing sexuality. Future collaboration with these older adults could inform the development of specific resources and shape service delivery to support the sexual needs of diverse populations. Asexual participants reported less positive and supportive therapeutic relationships, and further research is needed as they did not describe their experiences through the open questions.

The older adults’ rich descriptions provided insight into the impacts of physical and social changes, such as health concerns and partnership losses on psychological health and wellbeing confirming findings from Lyons et al. (2017) that changes in sexual function were not always considered the most distressing component. More important to participants’ health and wellbeing was the flow-on effect on roles in their relationships or the impact on their self- identity. This study addressed the need to understand and more appropriately support physical and social changes less in terms of direct limitations to sex, but instead recognize how these affect self-concept, confidence, and relationship roles. The need for occupational therapists to utilize biopsychosocial assessment and intervention methods was highlighted by the changes described to older adults’ sexuality roles, practices, intimacy, and self-identity.

The positive influence that relationships had on sexual ageing was evident, many partnered older adults did not see changes in their relationship and life roles as having a negative impact on their sexual identity or participation, confirming findings from Tetley et al. (2018). Long-term partnered adults overcame challenges by sharing and evolving their roles and practices, with evolving forms of companionship centering their sexual relationships. Newly partnered and re-partnered participants described discovering new roles and valued relationships as a means of overcoming negative experiences and re-engaging in intimacy. The study illustrated the important function relationships with shared values play toward continuing sexuality and provided insights into the potential impact of not having these experiences or grieving the loss of such relationships.

It was expected unpartnered older adults might experience poorer sexual outcomes, as they were described as experiencing increased ageism, and decreased sexual participation (Heywood et al., 2019; Lindau et al., 2007). Individuals not in relationships were more inclined to report less sexual activity but were not necessarily dissatisfied with their sexuality. Consistent levels of satisfaction across respondents may be explained by more sexually progressive views on sexuality, which saw notions of companionship and intimacy in the participation in mundane occupations as a driving contributor to positive sexuality. This finding is an important reminder to occupational therapists, and other service providers who changes to sexuality with ageing are not inherently negative, they are individualized and for some can be very rewarding.

Literature focuses on ageism and attitudes and portrays decreasing sexual interest, engagement, and participation (Heywood et al., 2019; Lyons et al., 2017). Participants reported mixed views about the impact of ageism. Participants across all demographics overwhelmingly disagreed that societal attitudes affected their sexuality, but they reported that they often did not seek help as they did not anticipate it would be useful. These findings demonstrate a sense of resilience, and growing awareness of self, which among the participants may have led to less sexual inhibitions.

Older adults who deliberately sought to overcome past and present negative experiences through openness, communication, and knowledge building were more likely to report positive sexuality, confirming findings from earlier studies (Fileborn et al., 2017c; Ménard et al., 2015). This study revealed that tangible barriers were more commonly experienced by individuals who were renting. In alignment with social determinants of health literature, it is reasonable to assume that socioeconomic position would affect help-seeking, and sexual health outcomes beyond what was controllable by the individual (AIHW, 2016). Environmental and social considerations must be addressed when negotiating therapists and other health providers are working with and creating resources for older adults’ sexuality. Various approaches to delivering resources and supports (online resources and pamphlets in health and community centers) may promote accessibility for this population.

A further implication of this study highlights the need for occupational therapists and HCPs to develop open communication, multidimensional assessment competencies, and holistic knowledge of sexual practices and supports to effectively address sexuality for older adults. The older adults largely self-explored changing dynamics in their relationships, changes to identity, understanding grief, loss, alternative therapies, and medicines. These findings show that service providers need to address similar diverse psychological, social, and health considerations when working with older adults. Future research is needed to support the development of holistic measures of sexuality for older adults to ensure sexuality is accurately addressed in research and practice. The development of standardized resources and assessments with the collaboration of consumers will ensure multifaceted components of sexuality, and the diversity of needs are addressed.

Consistency across the older adults’ experiences and desired support provisions highlight how occupational therapy and services could be used to promote positive sexual health across biopsychosocial domains throughout ageing. Centering practice around ideas of sexuality as an occupation with ever-changing identities, roles, and activities will promote occupational therapists to focus more on explorative interventions (which may vary to include assisting older adults to physically engage in sexual practices through assistive devices, assisting a client to utilize technology for online dating purposes).

Specialist sexuality services for older adults were depicted as being non-existent or inaccessible by some participants, though many agreed they would be beneficial. Further research is needed to support the implementation of age-specific sexual health centers and services.

## Limitations

The sample size of 75 older adults was smaller than anticipated and affected the statistics that could be examined and the generalizability of the results. However, in-depth experiences and understandings were illustrated in the qualitative responses. As a population-based sampling method was not used, most of the participants had similar demographic factors, which may have contributed to a repeated narrative across the findings. Demographic information, which included religious participation (past/present), ethnicity, and socioeconomic status, could have been collected from participants to examine how these factors influence sexuality norms, meaning, barriers, and facilitators. Older adults in the study were more open to discuss their sexuality, given their voluntary participation, which may detail why respondents showed a liberality in their attitudes, had good understandings of sexuality, and showed resilience toward societal attitudes. It is not known if this accurately represents the wider population.

## Conclusion

This study detailed the importance of sexuality to older adults, and it was understood and lived in varied ways, in contrast to often reported misconceptions that sexuality and practices cease due to ageing. Life-long experiences influenced the fluidity of sexuality, health, and wellbeing, and older adults remained connected to their sexual identity and desire as they aged.

These results reiterate that occupational therapists need to develop assessment skills and intervention knowledge to address diverse needs, and better support older adults’ values through open discussion, addressing psychological, wellbeing, social, and sexual health conditions as part of everyday practice.

**Key findings**

- Diverse experiences and personal factors influenced older adults’ engagement, satisfaction, and connection with their sexuality.
- Participants want more open, holistic, and comprehensive approaches to sexuality from occupational therapists and healthcare supports.

**What the study has added**

The study highlighted the need for inclusive governmental policies and initiatives that support sexuality and sexual health for older adults, and developments in healthcare organizations and professionals approaches to improve the provision of sexual supports.

## References

[bibr1-03080226231164277] Australian Institute of Health & Welfare (AIHW) (2016) Australia’s health 2016: Social determinants of health. Available at: https://www.aihw.gov.au/getmedia/11ada76c-0572-4d01-93f4-d96ac6008a95/ah16-4-1-social-determinants-health.pdf.aspx

[bibr2-03080226231164277] Alba B Lyons A Waling A , et al. (2020) Older lesbian and gay adults’ perceptions of barriers and facilitators to accessing health and aged care services in Australia. Health & Social Care in the Community 29: 918–927. 32761706 10.1111/hsc.13125

[bibr3-03080226231164277] AOTA (2008) Occupational therapy practice framework: Domain and process. American Journal of Occupational Therapy 62: 625–683. 10.5014/ajot.62.6.625 19024744

[bibr4-03080226231164277] Braun V Clarke V (2019) Reflecting on reflexive thematic analysis. Qualitative Research in Sport, Exercise and Health 11: 589–597.

[bibr5-03080226231164277] Chen YH Jones C Osborne D (2017) Exploratory study of Australian aged care staff knowledge and attitudes of later life sexuality. Australasian Journal on Ageing 36(2): E35–E38. 10.1111/ajag.12404 28319312

[bibr6-03080226231164277] DeLamater J Koepsel E (2015) Relationships and sexual expression in later life: A biopsychosocial perspective. Sexual and Relationship Therapy 30(1): 37–59. 10.1080/14681994.2014.939506

[bibr7-03080226231164277] Fileborn B Thorpe R Hawkes G , et al. (2015) Sex, desire and pleasure: Considering the experiences of older Australian women. Sexual and Relationship Therapy 30: 117–130. 10.1080/14681994.2014.936722 PMC4270421 25544829

[bibr8-03080226231164277] Fileborn B Hinchliff S Lyons A , et al. (2017a) The importance of sex and the meaning of sex and sexual pleasure for men aged 60 and older who engage in heterosexual relationships: Findings from a qualitative interview study. Archives of Sexual Behavior 46: 2097–2110. 28299563 10.1007/s10508-016-0918-9

[bibr9-03080226231164277] Fileborn B Lyons A Heywood W , et al. (2017b) Talking to healthcare providers about sex in later life: Findings from a qualitative study with older Australian men and women. Australasian Journal on Ageing 36: E50–E56. 28639430 10.1111/ajag.12450

[bibr10-03080226231164277] Fileborn B Lyons A Hinchliff S , et al. (2017c) Improving the sexual lives of older Australians: Perspectives from a qualitative study. Australasian Journal on Ageing 36: E36–E42. 28374505 10.1111/ajag.12405

[bibr11-03080226231164277] Fileborn B Lyons A Hinchliff S , et al. (2017d) Learning about sex in later life: Sources of education and older Australian adults. Sex Education 17: 165–179.

[bibr12-03080226231164277] Freak-Poli R Malta S (2020) An overview of sexual behaviour research in later life—Quantitative and qualitative findings. Australasian Journal on Ageing 39: 16–21. 10.1111/ajag.12773 32567181

[bibr13-03080226231164277] Health.Vic. (2020) Senior Rights Victoria. Available at: https://www2.health.vic.gov.au/ageing-and-aged-care/wellbeing-and-participation/preventing-elder-abuse/seniors-rightsvictoria#:~:text=Seniors%20Rights%20Victoria%20can%20help,to%20elder%20abuse%20and%20ageing (accessed 21 September 2021).

[bibr14-03080226231164277] Heywood W Minichiello V Lyons A , et al. (2019) The impact of experiences of ageism on sexual activity and interest in later life. Ageing & Society 39: 795–814.

[bibr15-03080226231164277] Hinchliff S Carvalheira AA Štulhofer A , et al. (2019) Seeking help for sexual difficulties: Findings from a study with older adults in four European countries. European Journal of Ageing 17: 185–195. 32547347 10.1007/s10433-019-00536-8 PMC7292843

[bibr16-03080226231164277] Hyland A McGrath M (2013) Sexuality and occupational therapy in Ireland–a case of ambivalence? Disability and Rehabilitation 35(1): 73–80. 10.3109/09638288.2012.688920 22657159

[bibr17-03080226231164277] IBM (2020) IBM SPSS Statistics. Available at: https://www.ibm.com/au-en/products/spss-statistics (accessed 13 May 2021).

[bibr18-03080226231164277] Laumann EO Glasser DB Neves RCS , et al. (2009) A population-based survey of sexual activity, sexual problems and associated help-seeking behavior patterns in mature adults in the United States of America. International Journal of Impotence Research 21: 171–178. 19242482 10.1038/ijir.2009.7

[bibr19-03080226231164277] Lindau ST Schumm LP Laumann EO , et al. (2007) A study of sexuality and health among older adults in the United States. New England Journal of Medicine 357: 762–774. 17715410 10.1056/NEJMoa067423 PMC2426743

[bibr20-03080226231164277] Lodge AC Umberson D (2012) All shook up: Sexuality of mid-to later life married couples. Journal of Marriage and Family 74: 428–443. 22904574 10.1111/j.1741-3737.2012.00969.x PMC3418692

[bibr21-03080226231164277] Lynch C Fortune T (2019) Applying an occupational lens to thinking about and addressing sexuality. Sexuality and Disability 37: 145–159.

[bibr22-03080226231164277] Lyons A Heywood W Fileborn B , et al. (2017) Sex, age & me: A national study of sex, sexual health and relationships among older Australians. Available at: https://www.latrobe.edu.au/data/assets/pdf_file/0009/788355/Sex-Age-and-Me-Broadsheet-20170817.pdf (accessed 18 September 2021). 10.1080/13691058.2017.1288268 28276921

[bibr23-03080226231164277] Ménard AD Kleinplatz PJ Rosen L , et al. (2015) Individual and relational contributors to optimal sexual experiences in older men and women. Sexual and Relationship Therapy 30: 78–93.

[bibr24-03080226231164277] Nowell LS Norris JM White DE , et al. (2017) Thematic analysis: Striving to meet the trustworthiness criteria. International Journal of Qualitative Methods 16: 1–13.

[bibr25-03080226231164277] Portney L Watkins M (eds) (2014) Surveys and questionnaires. In: Foundations of Clinical Research, 3rd edn. Philadelphia, Pennsylvania: Prentice-Hall Inc., pp.325–340.

[bibr26-03080226231164277] Qualtrics software (2019) Qualtrics and all other Qualtrics product or service names are registered trademarks or trademarks of Qualtrics, Provo, UT, USA. Available at: https://www.qualtrics.com (accessed 10 March 2021).

[bibr27-03080226231164277] Rahn A Jones T Bennett C , et al. (2020) Baby boomers’ attitudes to maintaining sexual and intimate relationships in long-term care. Australasian Journal on Ageing 39: 3–5. 10.1111/ajag.12732 32567184

[bibr28-03080226231164277] Sakellariou D Algado SS (2006) Sexuality and disability: A case of occupational injustice. British Journal of Occupational Therapy 69(2): 69–76. https://doi.org/10.1177%2F030802260606900204

[bibr29-03080226231164277] Sandberg L (2013) Just feeling a naked body close to you: Men, sexuality and intimacy in latewr life. Sexualities 16: 261–282.

[bibr30-03080226231164277] Schaller S Traeen B Lundin Kvalem I (2020) Barriers and facilitating factors in help-seeking: A qualitative study on how older adults experience talking about sexual issues with healthcare personnel. International Journal of Sexual Health 32: 65–80.

[bibr31-03080226231164277] Schofield MJ Forrester-Knauss C (2017) Surveys and questionnaires in health research. In: Liamputtong P (ed.) Research Methods in Health: Foundations for Evidence-Based Practice, 3rd ed. Oxford University Press, pp. 236–250.

[bibr32-03080226231164277] Sowa A Tobiasz-Adamczyk B Topór-Mądry R , et al. (2016) Predictors of healthy ageing: public health policy targets. BMC Health Services Research 16: 441–453. 27609315 10.1186/s12913-016-1520-5 PMC5016728

[bibr33-03080226231164277] Stahl KAM Gale J Lewis DC , et al. (2019) Pathways to pleasure: Older adult women’s reflections on being sexual beings. Journal of Women & Ageing 31: 30–48. 10.1080/08952841.2017.1409305 29210621

[bibr34-03080226231164277] Syme ML Cohn TJ Stoffregen S , et al. (2019) “At my age. . .”: Defining sexual wellness in mid-and later life. The Journal of Sex Research 56: 832–842. 29668312 10.1080/00224499.2018.1456510

[bibr35-03080226231164277] Tetley J Lee DM Nazroo J , et al. (2018) Let’s talk about sex–what do older men and women say about their sexual relations and sexual activities? A qualitative analysis of ELSA Wave 6 data. Ageing & Society 38: 497–521.

[bibr36-03080226231164277] United Nations, Department of Economic and Social Affairs, Population Division (UN) (2017) World Population Ageing 2017. Available at: https://www.un.org/en/development/desa/population/publications/pdf/ageing/WPA2017_Report.pdf (accessed 5 August 2021).

[bibr37-03080226231164277] von Humboldt S Low G Leal I (2020) Are older adults satisfied with their sexuality? Outcomes from a cross-cultural study. Educational Gerontology 46: 284–293.

[bibr38-03080226231164277] White CB (1982) A scale for the assessment of attitudes and knowledge regarding sexuality in the aged. Archives of sexual Behavior 11: 491–502. 7159218 10.1007/BF01542474

[bibr39-03080226231164277] World Health Organization. (2006). Defining sexual health: report of a technical consultation on sexual health, 28–31 January 2002, Geneva. World Health Organization. https://www.who.int/reproductivehealth/publications/sexual_health/defining_sexual_health.pdf?ua=1 (accessed 10 March 2021).

